# Selective disappearance based on navigational efficiency in a long‐lived seabird

**DOI:** 10.1111/1365-2656.14231

**Published:** 2025-01-27

**Authors:** Joe Wynn, Nathalie Kürten, Maria Moiron, Sandra Bouwhuis

**Affiliations:** ^1^ Institute of Avian Research Wilhelmshaven Germany; ^2^ School of Environmental Sciences University of Liverpool Liverpool UK; ^3^ Department of Evolutionary Biology Bielefeld University Bielefeld Germany

**Keywords:** behaviour, cognition, geolocation, learning, navigation, seabirds, selection

## Abstract

1. Whilst efficient movement through space is thought to increase the fitness of long‐distance migrants, evidence that selection acts upon such traits remains elusive. Here, using 228 migratory tracks collected from 102 adult breeding common terns (*Sterna hirundo*) aged 3–22 years, we find evidence that older terns navigate more efficiently than younger terns and that efficient navigation leads to a reduced migration duration and earlier arrival at the breeding and wintering grounds.
2. We additionally find that the age‐specificity of navigational efficiency in adult breeding birds cannot be explained by within‐individual change with age (i.e. learning), suggesting the selective disappearance of less navigationally efficient individuals.
3. This suggests that, at least in common terns, learning of navigational skills may be largely absent in adulthood, and limited to the pre‐breeding phase of life where tracking is more difficult.
4. We propose that selection might explain parts of the age‐specificity of navigational performance observed in migratory taxa more generally; discuss the causes and evolutionary implications of variation in navigational traits and the selective agents acting upon them; and highlight the necessity of longitudinal studies when considering changes in behaviour with age.

## Graphical abstract

Changes in migratory performance with age are typically assumed to reflect within‐individual learning processes. Here, we show that changes the navigational efficiency in a long‐lived seabird instead reflect the selective disappearance of inefficient individuals.

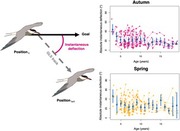

## 1 INTRODUCTION

Understanding how migratory trajectories are constructed and inherited, and how these processes interact with (and are shaped by) the environment, is essential when considering the ecology and evolution of long‐distance migrants. Among birds, migratory routes are thought to in‐part rely on (epi)genetically inherited information (Delmore & Irwin, 2014; Helbig, 1991), typically (though not universally; Thorup et al., 2020) thought to comprise a ‘clock and compass’; a clock to give migratory duration, and a compass to encode direction (Perdeck, 1958; Thorup et al., 2007; Yoda et al., 2017). In certain species, there also exists evidence for the cultural inheritance of information via social learning, with inexperienced navigators refining a genetically inherited route through social learning involving experienced conspecifics (Abrahms et al., 2021; Byholm et al., 2022; Chernetsov et al., 2004; Harrison et al., 2010; Mueller et al., 2013; Palacin et al., 2011; Rotics et al., 2016).

In addition to cultural and genetic inheritance, asocial learning—improvements in performance facilitated by information gained from experience—can also contribute to an individual's ability to navigate. Such learning is thought to play a role in the surprisingly precise philopatry observed in most migratory birds (Newton & Brockie, 2008), with pre‐migratory learning of specific gradient cue values—cue values that vary predictably through space such that specific values denote specific locations—thought to allow juvenile birds to pinpoint their breeding sites with remarkable accuracy (Baker, 1978; Wynn et al., 2022; Wynn, Padget, et al., 2020). Later in life, a substantial amount of the spatial information used by experienced navigators is thought to be learnt as well (for a review, see Åkesson et al., 2021), with the ability to compensate for both wind drift (Thorup et al., 2003; Wynn, Collet, et al., 2020) and artificial displacement (Chernetsov et al., 2017; Perdeck, 1958; Thorup et al., 2007) seemingly contingent upon experience. There is also increasing evidence that learnt improvements in navigational ability underpin the incremental ‘exploration‐refinement’ of the migratory route, with migratory efficiency (i.e. reduction in time and/or energetic costs of migration), destination, route, duration and timing all refined over successive attempts via associative learning (Abrahms et al., 2021; Campioni et al., 2020; Fayet et al., 2016; Guilford et al., 2011; Sergio et al., 2014; Verhoeven et al., 2021; Wynn et al., 2021).

**FIGURE 1:**
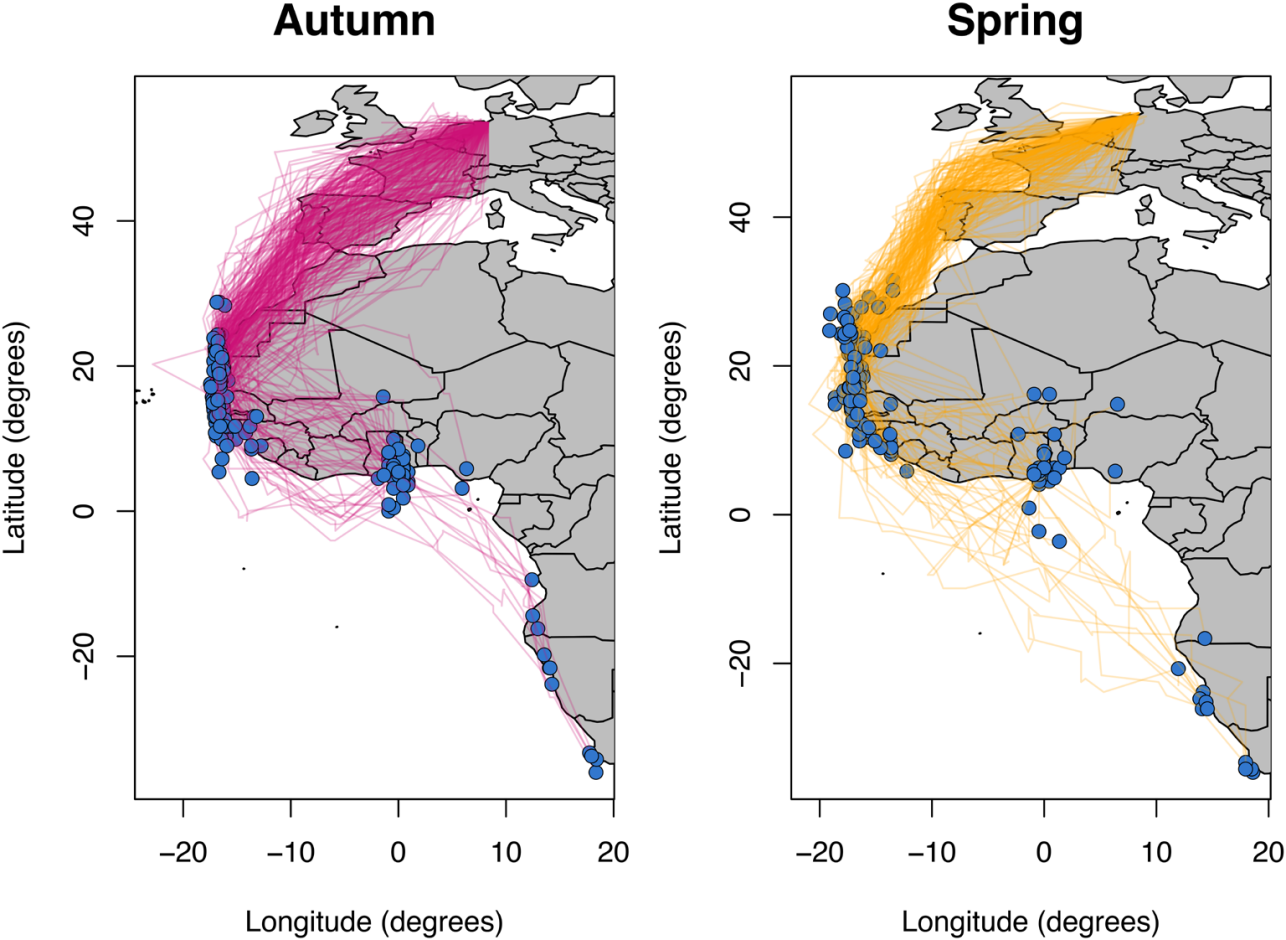
Adult, breeding common tern migratory routes and destinations. Migratory routes of common terns ages 3–22 years migrating in (left) autumn and (right) spring. Blue points mark the point identified as the arrival at, or departure from, the wintering site, respectively.

Within‐individual refinement of the migratory route is not, however, the only reason why we might expect older individuals to display improved/refined migratory phenotypes. Selective disappearance, owing to the mortality of less navigationally efficient individuals from the population (Maille & Schradin, 2016; Sergio et al., 2014, 2022; ‘selection’), would give much the same pattern. Separating the effects of selective disappearance from within‐individual learning is, therefore, crucial. At the same time, this is challenging, especially in the context of migratory efficiency, as it requires extensive longitudinal tracking data (i.e. collecting repeated data over multiple years) from birds of known age. Here, we present such data obtained by geolocator‐tracking breeding common terns (*Sterna hirundo*) aged 3–22 years (Figure 1), and investigate whether (i) navigational performance in adulthood is age‐specific and, if so, (ii) whether the age effect is best explained by learning or by the selective disappearance of birds based on their navigational phenotype.

## 2 MATERIALS AND METHODS

### 2.1 Study population

The common tern is a long‐lived, migratory seabird (Becker & Ludwigs, 2004). The data we present here come from a long‐term study population of common terns located at the Banter See in Wilhelmshaven on the German North Sea coast (53°30′40″ N, 08°06′20″ E). Here, chicks have been ringed since 1980, while the presence and reproductive performance of individually marked adults has been monitored following a standard protocol since 1992. Hereto, 101 adult birds were caught and marked with individually numbered subcutaneously injected transponders, and, since 1992, all locally hatched birds have been ringed as well as marked with such a transponder shortly prior to fledging.

As part of the standard protocol, arrival date is monitored using antennae affixed to the walls of the colony (Moiron et al., 2024) and the colony site is checked three times a week to find nests and record laying dates, clutch sizes and egg sizes. During incubation, which is shared between partners, antennae that can read the individual transponder codes are placed around each nest to identify breeding individuals. Combined with further nest‐checks to establish hatching, mark offspring and assess their growth and survival, these methods enable the documentation of individual life‐history trajectories (e.g. Moiron et al., 2022; Zhang et al., 2015).

### 2.2 Tracking

Between mid‐May and early July 2016–2021, 228 tracks were recorded from 102 birds (2016 *n* = 24 (22 retrieved); 2017 *n* = 36 (28 retrieved); 2018 *n* = 50 (40 retrieved); 2019 *n* = 54 (48 retrieved); 2020 *n* = 65 (54 retrieved); 2021 *n* = 54 (36 retrieved); total retrieval rate of geolocators with data = 80.5%) were equipped with an archival light‐level geolocator (Intigeo‐C65, Migrate Technology, UK) attached to the leg using a 10 mm aluminium ring. The total mass of the ring, glue and geolocator was 1.6 g, which equalled 1.2% ± 0.1 SD of the body mass of the birds at capture, and did not have a detectable effect on behaviour, reproductive performance or survival (Kürten et al., 2019). The geolocators were set to sample ambient light intensity every minute, with the maximum light intensity being stored every five minutes (‘mode 10’ on Migrate Technology devices).

### 2.3 Track processing

Upon return from migration, i.e. between mid‐May and early July 2017–2022, birds were retrapped to retrieve their geolocators carrying the archived light‐level data, from which their migratory trajectories were estimated using the R‐package ‘FLightR’ (Rakhimberdiev et al., 2017). Device failure mid‐trip notwithstanding, we have both a spring and autumn trajectory from each bird (in total: 228 tracks of 102 individuals, with 228 autumn and 184 spring trajectories).

To analyse these trajectories, we extracted two daily positions using the ‘run.particle.filter’ function and used the ‘stationary.migration.summary’ function to detect individual‐specific migration periods based on the dates of arrival to, and departure from, the breeding colony and wintering area. In essence, this involved using the posterior probability of movement for each day's calculated position to ascertain whether the bird was likely to be moving (movement probability >0.4). Based on these probabilities, geolocator fixes were then assembled into periods of movement and non‐movement, from which the breeding and wintering periods could be calculated. Spring and autumn migratory periods were defined as the intervening periods (for more details, see Kürten et al., 2022).

Once the beginning and end of each migration period were estimated for each track, we sought to remove any stopovers, during which birds would not necessarily exert a navigational preference, by removing GLS fixes where the distance moved was estimated at <100 km between fixes (removing *n* = 3451 fixes). This was meant to ensure that all fixes included in our dataset described the behaviour of birds moving in directed flight, and robustness to the selected cut‐off value was tested via reanalysis with the cut‐off value set to 50 km (removing *n* = 4575 fixes; see Table S4).

Autumn migration occurred between the 24th of July and 15th of October, whilst spring migration occurred between the 10th of February and 9th of May, with birds moving from their colony in Northern Germany to sites distributed between West and Southern Africa (see Kürten et al., 2022). Common terns took remarkably direct migratory routes—characterised by relatively few stopovers—with autumn and spring migration lasting on average 17.95 (±14.60 days standard deviation) and 27.56 days (±18.94 days standard deviation), respectively.

### 2.4 Analysis of age‐specific navigational performance

Given that GLS positional estimates are inferred using light levels, they are inherently less accurate than those obtained using other positioning technologies (e.g. GPS). However, since heavier GPS devices have been associated with changes in at‐sea behaviour in similar taxa (e.g. Gillies et al., 2020), we assumed that GLS devices were best‐suited to our purpose. The error inherent to light‐level geolocation comes from device shading and/or electrical errors in the device itself, and this error can manifest in one of two ways: (i) noise—indiscriminately affecting all devices, increasing the number of false negatives (e.g. noise obscuring a correlation between GLS positional information and a predictor), or (ii) bias—impacting devices non‐randomly, causing false positives (e.g. weather‐induced device shading causing variation in positional estimate, leading to a spurious correlation between cloud cover and distance travelled; Lisovski et al., 2018).

To minimise the effect of noise, we used the FLightR algorithm to process our GLS data, as it is robust to equinox error and leads to substantially less overall error than more traditional thresholding methods (*c*. 250 km error per fix; Halpin et al., 2021; Rakhimberdiev et al., 2017). In addition, our large sample size (228 migratory tracks from 102 individual terns) secured statistical power. For positional error to manifest as bias and in turn affect our study, the causes of positional error (i.e. device shading/electrical error) would have to correlate with age. To assess whether such bias was present in our dataset (i.e. whether birds of a given age were biased in their positional estimates), we tested whether the positional estimates of known‐location birds varied with age. This we did by assessing whether longitude and latitude varied with age over the months of June and July—when all birds tracked were known to be at the breeding colony—using a linear mixed effects model (see below). Given that this wasn't the case (see Supporting Information), we assume our conclusions to be unaffected by bias.

Characterising navigational performance without knowing a bird's preferred migratory route a priori is challenging, since whilst many birds take a direct migratory route (e.g. Prochazka et al., 2018; Schmaljohann et al., 2012), some do not (e.g. Alerstam, 2001; González‐Solís et al., 2009; Guilford et al., 2009; Lisovski et al., 2021; Mellone et al., 2013). We chose to characterise navigational performance for each GLS fix of each tern as the instantaneous deflection between the instantaneous migratory trajectory (i.e. the direction the bird is currently going in) and the goal (Padget et al., 2018; Wynn, Collet, et al., 2020). As such, instantaneous deflection was calculated twice‐daily for each bird and did not assume that the bird in question was following the Great Circle route (“beeline”). Whilst it is true that ‘improvements’ in navigational performance with age might reflect differing motivations to navigate efficiently towards the goal, we reasoned this was the less likely explanation of any change in performance since all birds were established breeders and, at least in spring, under selection to arrive and breed early (Dobson et al., 2017; Moiron et al., 2024).

**FIGURE 2:**
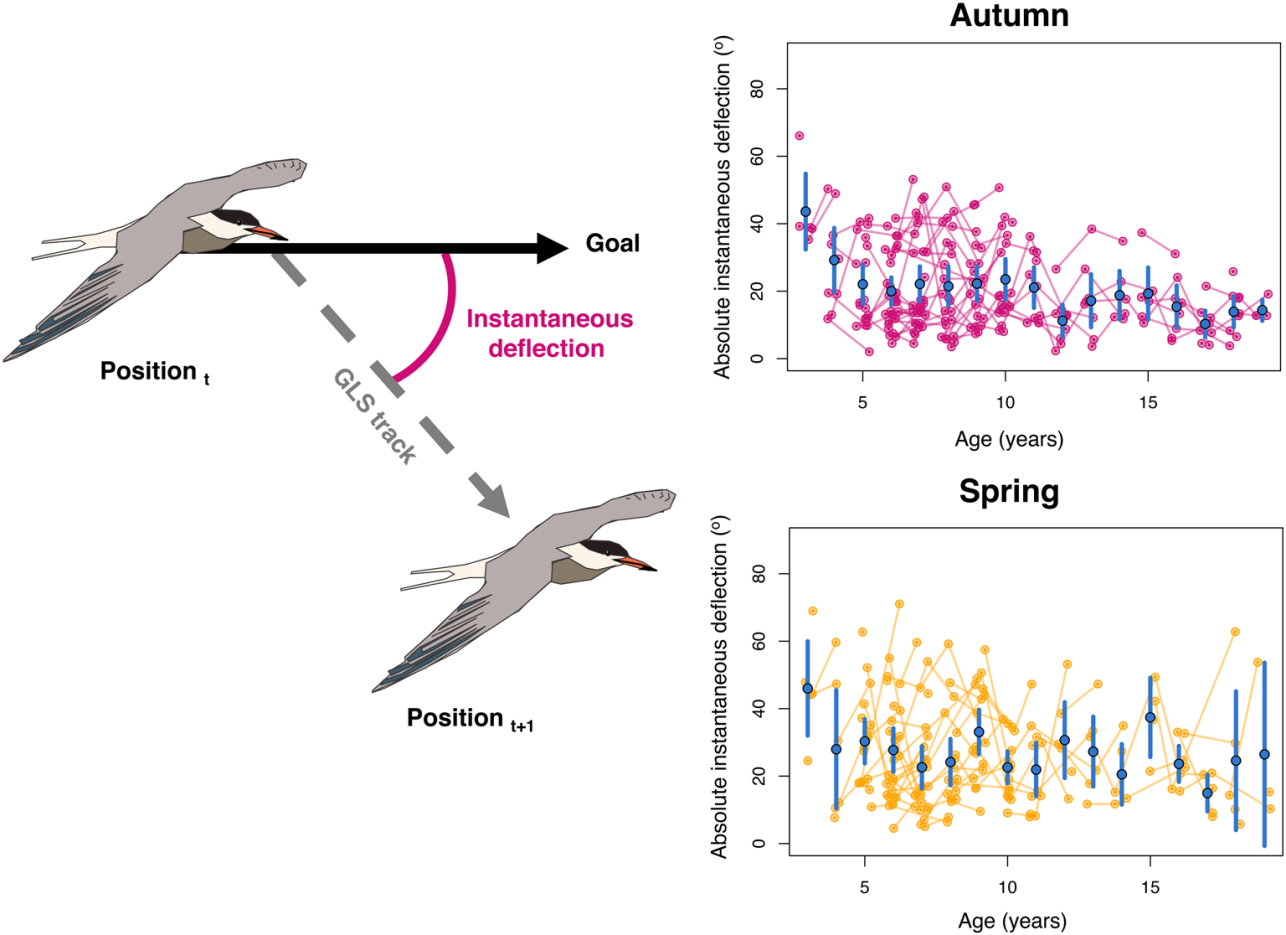
Age‐specific navigational performance in adult, breeding common terns. (left) A schematic detailing how instantaneous deflection is calculated from geolocator (GLS) tracks. (right; top) The mean instantaneous deflection from the goal for birds of different ages (in years) migrating in autumn and (right; bottom) spring. The blue points represent a mean deflection per age class, the error bars the associated 95% confidence interval. Each pink (autumn) or yellow (spring) point represents a single annual mean observation per individual, and each pink or yellow line links the average annual phenotype of repeatedly tracked birds. To ensure all data are visible, a small amount of jitter was added in the *x*‐axis to all points.

For autumn migration the destination was considered to be the highly repeatable individual‐specific wintering site (Kürten et al., 2022), whilst in spring the destination was considered to be the breeding site. Deflection angles were expressed as an absolute deflection between 0 and 180°, with 0° representing no difference between the bird's trajectory and the beeline between the observed position and the destination, and 180° meaning complete reversal (Figure 2). Whilst the response variable was angular, it was not circular, since the beginning and the end of the scale were not the same value. As such, we used linear rather than circular statistics to analyse our data.

To assess the effect of age on navigational performance, we used the R‐package ‘lme4’ (Bates et al., 2015) to run a linear mixed effects model with the GLS‐fix‐specific absolute instantaneous deflection as a response variable assuming a Gaussian error distribution, and with track identity, nested within individual identity, as random effects to account for pseudoreplication (Padget et al., 2018). Because multiple birds of different ages (expressed in years since hatching) were tracked per season—and no birds were kept in the study for its entire duration—year‐on‐year changes in migration caused by the environment were unlikely to confound with age in any analysis.

Age was partitioned into an ‘average age’ and ‘delta age’ component (van de Pol & Wright, 2009), whereby an individual's average age was defined as the average of all ages at which it was tracked, while delta age was defined as the difference between an individual's age for a given track and its average age (i.e. delta age = age − average age). When both were added as a covariate to the model, average age reflected whether birds tracked at different ages differed in their navigational performance (i.e. an among‐individual pattern), whilst delta age represented any within‐individual change with age (e.g. learning; van de Pol & Wright, 2009). A significant effect of delta age would suggest that learning (or senescence, depending on the direction of the effect) explained changes in navigational performance with age, whilst a significant difference between the effects of average age and delta age would indicate selective disappearance. A graphical illustration of this principle can be found in Figure S1. We also included the interaction between average age and delta age to test for non‐linear effects of age, because learning could decelerate as birds grow older. Finally, we added season as a 2‐level class variable (autumn and spring), both as a main effect and in interaction with all age components, in order to test whether (age‐specific) navigational performance differed between autumn and spring migration. Since both average age and delta age were on the same scale, and there were no other predictors, we did not mean‐centre or normalise the variable.

Significance was assessed using likelihood ratio tests, comparing the hypothesis model to a null model that was identical to the hypothesis model save for the exclusion of the interaction/term under investigation. Effect sizes and bootstrapped 95% confidence intervals, which were estimated using the R‐package ‘arm’, are reported in Table 1 and Table S1. The full model was simplified, allowing for the creation of a minimally adequate model with more immediately interpretable effect sizes. Since the three‐way interaction between season, average age and delta age; the two‐way interaction between average age and delta age; the two‐way interaction between season and average age; and the two‐way interaction between season and delta age were non‐significant, these terms were excluded during a step‐wise backwards elimination process (Table 1). The resulting minimally adequate model included the main effects of season, average age, and delta age. Delta age was included irrespective of significance so as the difference between the effects of the delta and average age components could be assessed. This we did by estimating the difference between both effects and assessing whether the associated bootstrapped 95% confidence intervals overlapped zero (van de Pol & Wright, 2009).

**TABLE 1:** Results from linear mixed‐effect models testing the effects of age and season on adult, breeding common tern navigational efficiency (estimated as the instantaneous deflection from the goal measured in degrees).

Effect type	Term	Effect size (^o^)	95% CI (^o^)	Chi‐squared	*p*‐value
Retained fixed effects	Intercept	27.750	23.215, 32.690	—	—
	Season = spring	9.740	6.977, 12.503	34.049	<0.001**
	Average age	−0.870	−1.335, −0.441	13.272	<0.001**
	Delta age	0.851	−0.367, 2.359	2.190	0.138
Random effects	Individual identity	63.264	47.728, 80.689	—	—
	Track identity (nested within individual identity)	6.316	5.141, 7.625	—	—
	Residual	1139.896	1092.661, 1189.074	—	—
Rejected fixed effects	Average age × delta age	−0.251	−0.809, 0.259	0.258	0.611
	Average age × delta age × season	0.187	−0.583, 0.926	1.326	0.243
	Season spring × average age	0.171	−0.412, 0.754	0.437	0.507
	Season = spring × delta age	0.793	−1.378, 3.295	0.071	0.790

*Note*: 95% confidence intervals are calculated for each fixed and random effect using bootstrapping (see Section 2).

* Indicates significance of *p* < 0.05.

** Indicates significance of *p* < 0.01.

We proceeded to re‐run all analyses using only the 177 tracks (i.e. 78% of all tracks) that wintered in West Africa. We did so because birds migrating further south seemingly followed different, less efficient routes (see Figure 1), such that any difference in navigational performance might represent differences in destination rather than performance (see Tables S1 and S2).

### 2.5 Analysis of the effect of navigational efficiency on migratory timing and duration

To test whether the duration of migration, or the resulting arrival date (known to be under selection, at least in spring; see Moiron et al., 2024), varied with instantaneous deflection, for each route we regressed the overall time spent migrating (i.e. the time of the end of migration minus the time of migration onset, in days; assuming a Gaussian error distribution) or the Julian date of arrival against the average instantaneous deflection observed over the route, including individual identity as a random effect and season as a fixed effect.

As above, significance was assessed using likelihood ratio tests comparing the hypothesis model to a null model that was identical to the hypothesis model save for the exclusion of the interaction/term under investigation. All effect sizes and bootstrapped 95% confidence intervals are reported in Table 2 and Table S2.

**TABLE 2:** Results from linear mixed‐effect models testing the correlation between absolute deflection and migration timing and duration (measured in Julian day and days, respectively).

Effect type	Term	Julian arrival date				Migratory duration (days)			
		Effect size (days)	95% CI (days)	Chi‐squared	*p*‐value	Effect size (days)	95% CI (days)	Chi‐squared	*p*‐value
Fixed effects	Intercept	256.612	252.798, 260.449	—	—	9.618	6.419, 12.726	—	—
	Deflection	0.223	0.088, 0.359	3.900	0.048*	0.173	0.0851, 0.267	5.180	0.023*
	Season = spring	−146.507	−151.352, −141.541	1407.600	<0.001**	3.403	0.355, 6.817	4.211	0.040*
	Season = spring × deflection	−0.211	−0.381, −0.055	6.279	0.012*	−0.143	−0.248, −0.043	5.798	0.016*
Random effects	Individual identity	56.535	44.613, 70.617	—	—	75.438	61.992, 89.869	—	—
	Residual	61.400	53.805, 70.446			111.843	96.584, 129.057	—	—

*Note*: 95% confidence intervals are calculated for each fixed and random effect using bootstrapping (see Section 2).

* Indicates significance of *p* < 0.05.

** Indicates significance of *p* < 0.01.

## 3 RESULTS

Instantaneous deflection was on average 1.023° lower for each year of increase in average age (*χ* ^2^1 = 13.272, *p* < 0.001; Table 1); whereas the within‐individual effect of delta age was non‐significantly positive (which, if anything, would represent a within‐individual *reduction* in efficiency with age; Table 1; Figure 2). The effects of average and delta age significantly differed from one another (difference: −1.805°; 95% CI: −3.117°, −0.643°). We, additionally, found that there was no significant interaction between the average and delta age components (Table 1), implying that there was no detectable increase/decrease in the contribution of learning to the phenotype across the assessed ages (3–22 years; *χ* ^2^1 = 0.258, *p* = 0.611; Table 1).

We also tested whether birds undertaking autumn and spring journeys differed in navigational efficiency, and for an interaction between season and both age components. Whilst we found that birds deflected more and took a less direct route in spring compared to autumn (*χ* ^2^1 = 34.049, *p* < 0.001; Table 1), we found no significant difference in the effects of average or delta age between the seasons (Table 1).

To ensure that the observed effect was not an artefact of deflections from the beeline reflecting coast‐following behaviour in birds wintering south of West Africa (Figure 1 and Figure S2), we repeated our analysis with these birds removed. This gave a very similar coefficient for average age (−0.863, 95% CI: −1.335, −0.685), which was still significantly different from zero (*χ* ^2^1 = 13.012, *p* < 0.001; Table S1) and significantly different from the delta age component (difference: −1.765°; 95% CI: −3.335°, −0.340°), which itself remained non‐significantly positive (0.888, 95% CI: −0.671, 2.303, *χ* ^2^1 = 1.237, *p* = 0.266; Table S1).

**FIGURE 3:**
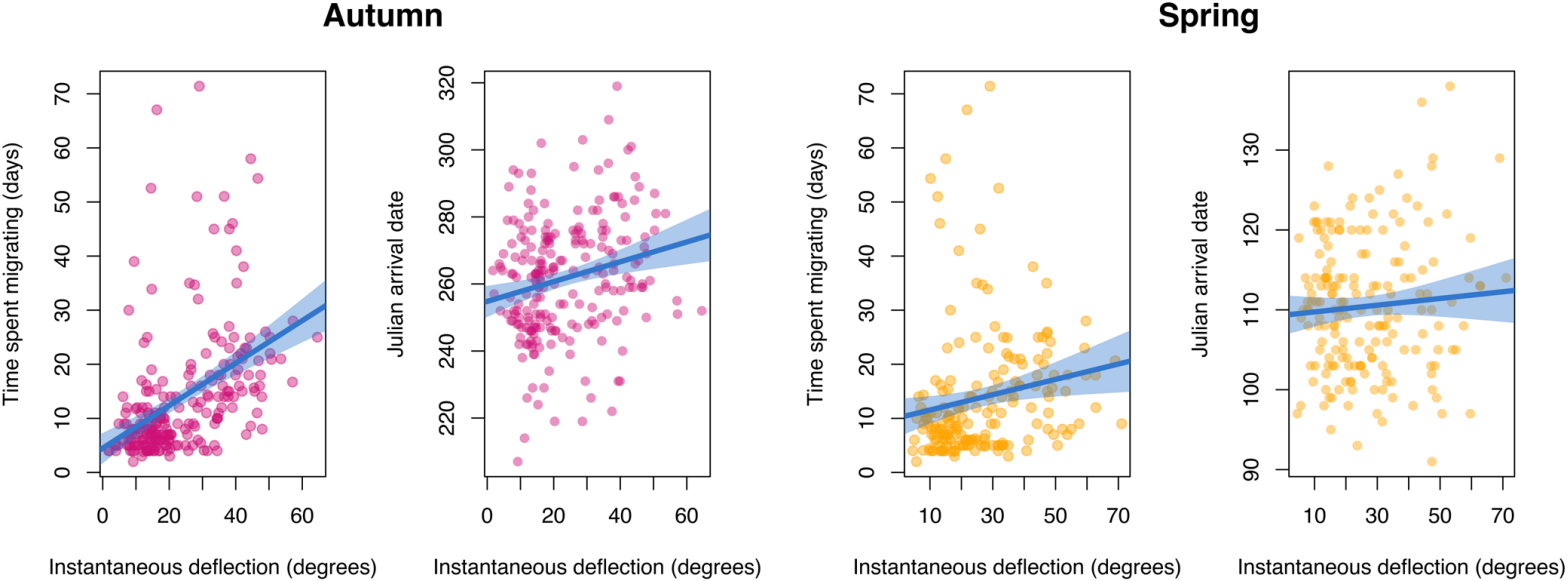
Correlations between common tern navigational performance and migration timing and duration. Migration duration and Julian arrival date plotted against instantaneous deflection in (left) autumn and (right) spring. In all plots the blue line represents a least means squares linear regression and the shaded area 95% confidence intervals. Each pink (autumn) or yellow (spring) point represents a single individual GLS track.

In addition to testing the age‐specificity of navigational proficiency, we also tested whether variation in navigational efficiency translated to variation in migratory duration. We indeed found a negative correlation between instantaneous deflection and the time spent migrating (*χ* ^2^1 = 5.180, *p* = 0.023; Figure 3, Table 2), although this relationship differed between the seasons (with the days per degree being smaller in spring than in autumn; *χ* ^2^1 = 6.515, *p* = 0.011; Figure 3, Table 2). We also found that arrival dates to the wintering and breeding areas were earlier with increased navigational efficiency (*χ* ^2^1 = 3.900, *p* = 0.049), with this effect again being more pronounced in autumn (*χ* ^2^1 = 6.279, *p* = 0.012; Figure 3).

## 4 DISCUSSION

Whilst there is an extensive and growing literature demonstrating the effects of learning on the navigational phenotype in young birds (e.g. Sergio et al., 2014; Wynn, Collet, et al., 2020), verification that learning continues throughout an individual's life is thus far limited. Using longitudinal data for adult breeding common terns, we here present evidence that older individuals navigate more efficiently than younger individuals, but, perhaps surprisingly, that within‐individual improvement in navigational performance in adulthood is unlikely to underlie this pattern. While in theory it is possible that this trend reflects selective emigration rather than mortality, we think this is unlikely given that no breeding birds from the Banter See have ever been found breeding elsewhere (Becker et al., 2008). This is in contrast to immatures, sightings of which at other colonies are much more common, suggesting that this apparent lack of emigration in adults is not an artefact of observation bias and hence that selective mortality based on navigational phenotype is a much more likely explanation of the pattern at hand.

What ‘selective mortality based on navigational efficiency’ actually means is unclear, and its importance to the evolution of navigational traits is necessarily contingent upon the source of any variance. Since the ability to orient through space is, in experienced individuals, thought to utilise a culturally or genetically inherited trajectory, which is augmented with, or replaced by, some sort of asocially learnt spatial memory (Chernetsov et al., 2017; Guilford & de Perera, 2017; Padget et al., 2019), it follows that the selective disappearance of less efficient individuals might act upon some part(s) of this process. For example, inefficient individuals with imperfect ‘maps’ of their migratory route would find it difficult to reorient following weather‐borne displacement, leading to direct selection based on the navigational mechanism. Alternatively, or additionally, selection might act upon among‐individual variance in learning proficiency (Morand‐Ferron, 2017), with an aptitude for learning navigational information early in life potentially reducing probabilities of mortality throughout life.

Given the lack of information on navigational behaviour during the early developmental stages of our tracked birds, differentiating between these hypotheses is at present impossible. Our results could even reflect selection based on navigational *performance* rather than *ability*, especially if navigational efficiency were impacted by extraneous influences (such as disease/nutrition). If this were true, selection acting upon susceptibility to such factors would appear similar to selection based upon navigational ability itself. Indeed, it is even possible that selection on navigational efficiency reflects selection *against* other behavioural traits, such as exploration. That said, since we find an effect of navigational efficiency on migratory duration and arrival date, with the latter known to be under selection in terns at least in spring (Moiron et al., 2024) this may be the mechanism via which selection might act. Given that reduced migratory duration, however, could result from other factors as well (e.g. reduced time at stopover sites), further investigation will be essential to ascertain whether selection acts upon navigation itself, or whether selective disappearance based on navigational phenotype instead reflects selection on an as yet unknown correlate.

Irrespective of what trait(s) selection acts upon precisely, for a trait to exhibit an evolutionary response to selection it must be repeatable and, ultimately, heritable. Using the random effect estimates from our model presented in Table 1, we calculated the individual repeatability of instantaneous deflection—which could be seen as the minimum heritability of the trait (since if a trait is not repeatable within an individual it cannot be inherited between individuals)—by dividing the among‐individual variance by the total phenotypic variance not attributable to fixed effects. This we found to be 6.0% (95% CI = 4.1%, 8.3%). As such, our estimate of individual repeatability is comparatively small when considered within the context of other estimates of behavioural repeatability (~40%; Bell et al., 2009; Holtmann et al., 2017), in turn suggesting that the selective disappearance reported here can only result in a slow evolutionary response, if at all. This is, however, necessarily conjecture, and understanding the factors that influence the inheritance and ontogeny of migratory behaviour remains a central question in the study of animal behaviour going forward.

Whilst many studies have found navigational performance to be age‐specific (Campioni et al., 2020; Thorup et al., 2003; Wynn, Collet, et al., 2020) and strongly informed by learning (Mueller et al., 2013; Sergio et al., 2014), few have attempted to determine whether age effects always reflect learning or might, in some cases, instead indicate the selective disappearance of unfit individuals (Sergio et al., 2022). Although drawn correlatively using natural variation, we suggest that our findings—(i) that older terns navigate more efficiently, and (ii) that this among‐individual pattern cannot be explained by within‐individual changes with age (i.e. learning) — show that selection represents an important driver of differences in navigational ability between age classes, at least in adulthood. More generally, our results highlight the necessity of longitudinal studies in differentiating between selective disappearance and ontogeny, both when considering navigation specifically and animal behaviour more generally.

## AUTHOR CONTRIBUTIONS

Conceptualisation: Joe Wynn, Sandra Bouwhuis; Methodology: Joe Wynn, Nathalie Kürten, Maria Moiron, Sandra Bouwhuis; Software: Joe Wynn, Nathalie Kürten, Maria Moiron; Analysis: Joe Wynn, Nathalie Kürten, Maria Moiron, Sandra Bouwhuis; Investigation: Joe Wynn, Nathalie Kürten, Maria Moiron, Sandra Bouwhuis; Data curation: Nathalie Kürten, Maria Moiron, Sandra Bouwhuis; Writing—original draft: Joe Wynn; Writing—review & editing: Joe Wynn, Nathalie Kürten, Maria Moiron, Sandra Bouwhuis; Supervision: Sandra Bouwhuis; Project administration: Sandra Bouwhuis; Funding acquisition: Sandra Bouwhuis, Nathalie Kürten.

## CONFLICT OF INTEREST STATEMENT

The authors declare no competing interests.

## ETHICAL APPROVAL

All work was carried out under licence from LAVES, permit numbers 33.19–42,502–04‐16/2128 (2016 pilot), 33.19–42,502–04‐17/2449 (2017) and 33.19–42,502–04‐19/3068 (2019–2023).

## Supporting information


**Figure S1.** Visualisation of our analytical framework based on van de Pol and Wright (2019).
**Figure S2.** Common tern tracks coloured by wintering destination.
**Figure S3.** Common tern bearing a Migrate Tech C65 geolocator (as used in our study).
**Table S1.** Results from linear mixed‐effect models testing the effects of age and season on adult common tern navigational efficiency (estimated as the instantaneous deflection from the goal) for birds wintering in West Africa only.
**Table S2.** Results from linear mixed‐effect models testing the correlation between absolute deflection and migratory phenology of common terns wintering in West Africa.
**Table S3.** Results from linear mixed‐effect models testing the effects of age and season on adult common tern navigational efficiency (estimated as the instantaneous deflection from the goal) for all birds with the movement threshold reduced from 100 km to 50 km (see main text).
**Table S4.** Results from linear mixed‐effect models testing the effects of age and on estimated longitude for birds where position was known and unmoving.
**Table S5.** Results from linear mixed‐effect models testing the effects of age and on estimated latitude for birds where position was known and unmoving.

## Data Availability

Data available from the Dryad Digital Repository: https://doi.org/10.5061/dryad.m63xsj4cj (Wynn et al., 2024).
