# Supplementary material for: Selective disappearance based on navigational efficiency in a long‐lived seabird
Source: J Anim Ecol. 2025 Jan 27;94(4):535–44. doi: 10.1111/1365-2656.14231 (PMC11962229; doi:10.1111/1365-2656.14231)
Supplement: Supplementary file 1 — Figure S1. Visualisation of our analytical framework based on van de Pol and Wright (2019). Figure S2. Common tern tracks coloured by wintering destination. Figure S3. Common tern bearing a Migrate Tech C65 geolocator (as used in our study). Table S1. Results from linear mixed‐effect models testing the effects of age and season on adult common tern navigational efficiency (estimated as the instantaneous deflection from the goal) for birds wintering in West Africa only. Table S2. Results from linear mixed‐effect models testing the correlation between absolute deflection and migratory phenology of common terns wintering in West Africa. Table S3. Results from linear mixed‐effect models testing the effects of age and season on adult common tern navigational efficiency (estimated as the instantaneous deflection from the goal) for all birds with the movement threshold reduced from 100 km to 50 km (see main text). Table S4. Results from linear mixed‐effect models testing the effects of age and on estimated longitude for birds where position was known and unmoving. Table S5. Results from linear mixed‐effect models testing the effects of age and on estimated latitude for birds where position was known and unmoving. [file JANE-94-535-s001.zip › Navigational efficiency supp matt JAE 031024.docx]

Supplementary material

to

**Selective disappearance based on navigational efficiency in a long-lived seabird**

by Joe Wynn^1*^, Nathalie Kürten^1^, Maria Moiron^1,2^ and Sandra Bouwhuis^1^

^1^Institute of Avian Research, An der Vogelwarte 21, D-26386 Wilhelmshaven, Germany^2^ ^2^Department of Evolutionary Biology, Bielefeld University, Bielefeld, Germany

*****Corresponding author: joseph.wynn@ifv-vogelwarte.de

*Assessing the age-specific effects of device shading*

We infer the main finding of our manuscript – that older birds navigate more efficiently than younger individuals, but that there is no within-individual change – using light-level geolocation. Given the mechanisms by which geographic location is calculated from light levels, ‘type one’ (false positive) error might seem unlikely. However, age-specific shading of the device caused by differences in behaviour (e.g. difference in roosting behaviour) could in principle cause differences in position accuracy that might, in turn, drive type one error. Whilst this would seem extremely unlikely, and we have no *a priori* reason to suspect this to be true, we nonetheless assessed whether this was the case.

We investigated the effects of age-specific shading by assessing differences in the calculated positions of birds when they were at a known site; if there was an effect of age on position when all birds were in the same place, we would know that there was age-based uncertainty in position. This we did by assessing the effects of average and delta age on the calculated longitude and latitude over the months of June and July, when birds were known to be at their colony in Wilhelmshaven (North Germany) using RFID tag detections (see main text methods). Based on this, we found that no effect of delta or average age on the perceived longitude and latitude of tagged common terns (see Tables S4 and S5). Based on this, we conclude that age-specific shading of the geolocator device is not driving positional uncertainty and, in turn, is unlikely driving type one error in our analyses.

**
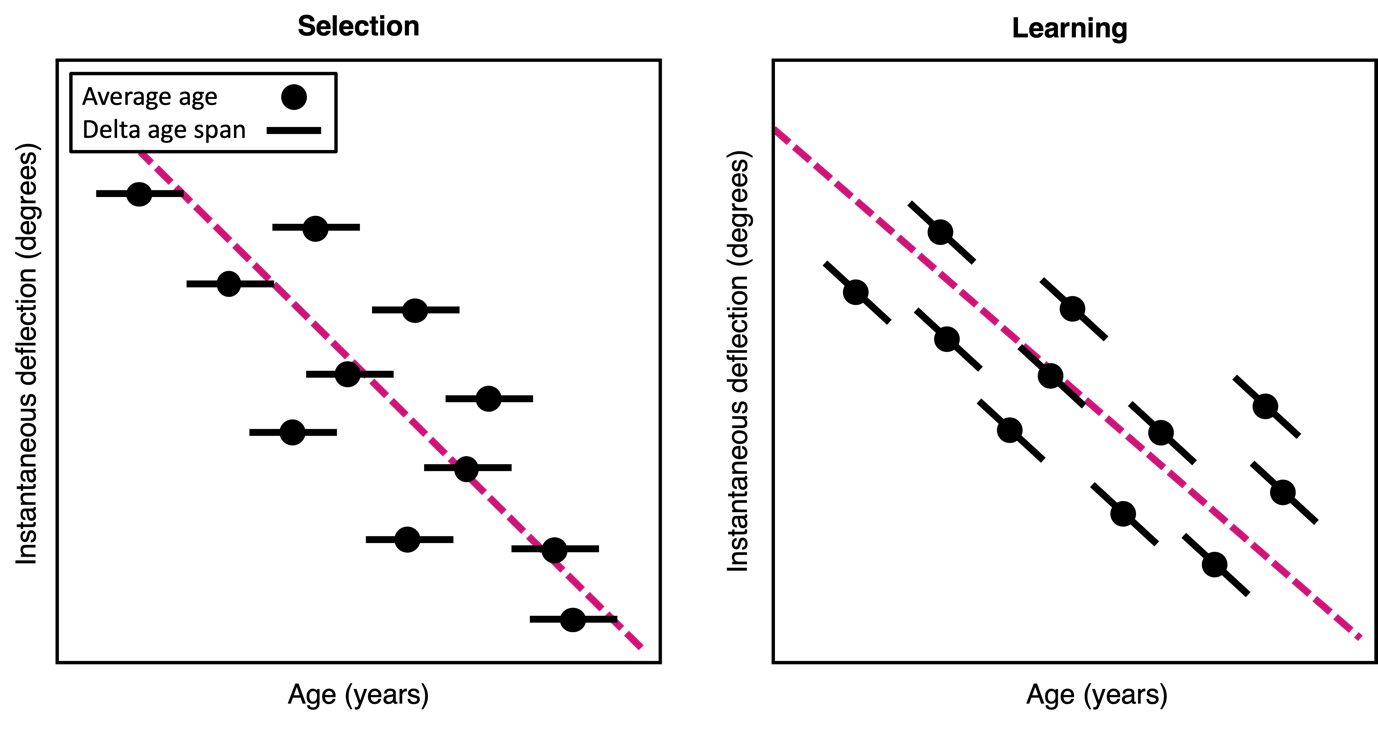
Figure S1: Visualisation of our analytical framework based on van de Pol and Wright (2019).** A schematic showing how differences between (left), or similarities in (right), delta age (the span shown by each black line) and average age (black circles) effects reflect selection (left) and learning (right) in navigational performance (the average effect of which is shown using the pink dotted line).

**Figure S2: Common tern tracks coloured by wintering destination.** Birds classified as likely to be wintering in West Africa – and hence those retained for reanalysis in tables S1 and S2 – are highlighted in pink, whilst those wintering outside of the Canary Current are highlighted in blue.

**Figure S3: Common tern** *
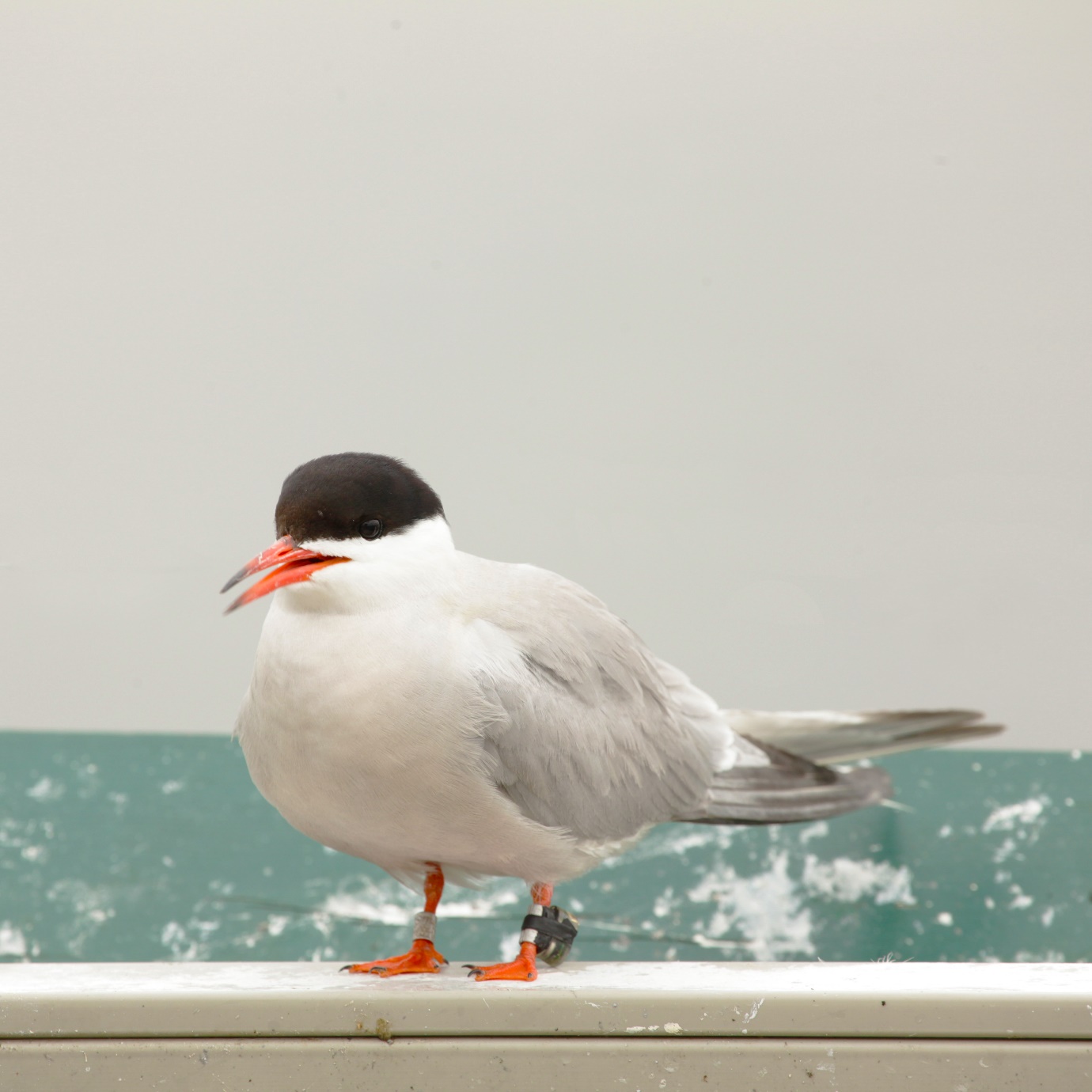
***bearing a Migrate Tech C65 geolocator (as used in our study).**

**Table S1: Results from linear mixed-effect models testing the effects of age and season on adult common tern navigational efficiency (estimated as the instantaneous deflection from the goal) for birds wintering in West Africa only.** 95% confidence intervals are calculated for each fixed and random effect using bootstrapping (see Methods).

| Effect  type | Term | Effect size (^o^) | 95%  CI (^o^) | Chi-  squared | p-  value |
| --- | --- | --- | --- | --- | --- |
| *Retained  fixed  effects* | Intercept | 27.652 | 22.751,  32.457 | - | - |
|  | season = spring | 9.727 | 6.928,  12.488 | **48.797** | **<0.001** |
|  | average age | -0.863 | -1.335,  -0.685 | **13.012** | **<0.001** |
|  | delta age | 0.888 | -0.671,  2.303 | 1.237 | 0.266 |
| *Random  effects* | individual identity | 63.461 | 47.578,  83.097 | - | - |
|  | track identity (nested within individual identity) | 6.304 | 5.191,  7.586 | - | - |
|  | residual | 1207.703 | 1149.550, 1273.677 | - | - |
| *Rejected  fixed effects* | average age * delta age | -0.275 | -0.872,  0.345 | 0.267 | 0.605 |
|  | average age * delta age * season | 0.324 | -0.445,  1.137 | 0.640 | 0.424 |
|  | season = autumn * average age | 0.277 | -0.369,  0.872 | 0.500 | 0.480 |
|  | season = spring * delta age | -1.962 | -10.542,  6.486 | 0.811 | 0.368 |

|  |  | Julian arrival date | | | | Migratory duration (days) | | | | |
| --- | --- | --- | --- | --- | --- | --- | --- | --- | --- | --- |
| *Effect type* | **Term** | **Effect size (days)** | **95% CI** | **Chi-squared** | **p-value** | **Effect size**  **(days)** | **95% CI (days)** | **Chi-squared** | | **p-value** |
| *Fixed effects* | Intercept | 255.243 | 251.154, 259.278 | - | - | 8.047 | 4.904, 11.116 | | - | - |
|  | deflection | 0.309 | 0.128, 0.505 | **4.364** | **0.037** | 0.278 | 0.141, 0.424 | | **7.635** | **0.006** |
|  | season = spring | -145.248 | -149.904, -140.181 | **1214.900** | **<0.001** | 4.279 | 0.587, 8.063 | | **5.309** | **0.021** |
|  | season = spring *deflection | -0.275 | -0.275,  -0.066 | **6.515** | **0.017** | -0.222 | -0.381,  -0.057 | | **6.881** | **0.009** |
| *Random effects* | individual identity | 55.121 | 42.299, 69.569 | - | - | 57.077 | 45.553, 70.345 | | - | - |
|  | residual | 117.114 | 100.909, 134.554 | - | - | 70.229 | 60.409, 80.944 | | - | - |

**Table S2: Results from linear mixed-effect models testing the correlation between absolute deflection and migratory phenology of common terns wintering in West Africa.** 95% confidence intervals are calculated for each fixed and random effect using bootstrapping (see Methods).

**Table S3: Results from linear mixed-effect models testing the effects of age and season on adult common tern navigational efficiency (estimated as the instantaneous deflection from the goal) for all birds with the movement threshold reduced from 100km to 50km (see main text).** 95% confidence intervals are calculated for each fixed and random effect using bootstrapping (see Methods).

| Effect  type | Term | Effect size (^o^) | 95%  CI (^o^) | Chi-  squared | p-  value |
| --- | --- | --- | --- | --- | --- |
| *Retained  fixed effects* | Intercept | 30.072 | 24.917,  35.086 | - | - |
|  | season = spring | 6.761 | 4.419,  9.080 | **32.556** | **<0.001** |
|  | average age | -0.827 | -1.297  -0.330 | **10.714** | **<0.001** |
|  | delta age | 0.780 | -0.321,  1.898 | 1.632 | 0.201 |
| *Random  effects* | individual identity | 0 | 0,  0 | - | - |
|  | track identity (nested within individual identity) | 98.871 | 76.314,  127.841 | - | - |
|  | residual | 1207.703 | 1149.550, 1273.677 | - | - |
| *Rejected  fixed effects* | average age * delta age | -0.281 | -0.783,  0.228 | 0.05 | 0.814 |
|  | average age * delta age * season | 0.445 | -0.290,  1.171 | 1.510 | 0.471 |
|  | season = autumn * average age | -3.457 | -10.950,  3.747 | 7.131 | 0.0673 |
|  | season = spring * delta age | 0.445 | -0.290,  1.171 | 1.82 | 0.603 |

**Table S4: Results from linear mixed-effect models testing the effects of age and on estimated longitude for birds where position was known and unmoving.** 95% confidence intervals are calculated for each fixed and random effect using bootstrapping (see Methods).

| Effect  type | Term | Effect size (^o^) | 95%  CI (^o^) | Chi-  squared | p-  value |
| --- | --- | --- | --- | --- | --- |
| *Fixed effects* | average age | 0.003 | -0.023,  0.029 | 0.506 | 0.822 |
|  | delta age | 0.0935 | -0.019,  0.194 | 2.60 | 0.112 |
| *Random  effects* | individual identity | 0.562 | 0.523,  0.5960 | - | - |

**Table S5: Results from linear mixed-effect models testing the effects of age and on estimated latitude for birds where position was known and unmoving.** 95% confidence intervals are calculated for each fixed and random effect using bootstrapping (see Methods).

| Effect  type | Term | Effect size (^o^) | 95%  CI (^o^) | Chi-  squared | p-  value |
| --- | --- | --- | --- | --- | --- |
| *Fixed effects* | average age | -0.001 | -0.033,  0.032 | 0.000 | 0.996 |
|  | delta age | 0.107 | -0.043,  0.1249 | 1.90 | 0.168 |
| *Random  effects* | individual identity | 0.562 | 0.523,  0.5960 | - | - |
